# Circular dorsal ruffles disturb the growth factor-induced PI3K-AKT pathway in hepatocellular carcinoma Hep3B cells

**DOI:** 10.1186/s12964-022-00911-6

**Published:** 2022-07-07

**Authors:** Xiaowei Sun, Yujie Liu, Shuheng Zhou, Li Wang, Jinzi Wei, Rui Hua, Zhongyang Shen, Sei Yoshida

**Affiliations:** 1 State Key Laboratory of Medicinal Chemical Biology, Frontiers Science Center for Cell Responses, College of Life Sciences, Nankai University, No. 94 Weijin Road, Tianjin, 300071 China; 2 Organ Transplant Department, Tianjin First Central Hospital, School of Medicine, Nankai University, Tianjin, China; 3 Research Institute of Transplant Medicine, Nankai University, Tianjin, China; 4 Tianjin Key Laboratory for Organ Transplantation, Tianjin, China

**Keywords:** Circular dorsal ruffles, AKT, PI3K, Hep3B

## Abstract

**Background:**

Circular dorsal ruffles (CDRs) are rounded membrane ruffles induced on the dorsal surfaces of cells stimulated by growth factors (GF). They can serve as signal platforms to activate AKT protein kinase. After GF stimulation, phosphatidylinositol 3-kinase (PI3K) generates phosphatidylinositol (3,4,5)-triphosphate (PIP3) in the plasma membrane. PIP3 accumulates inside CDRs, recruits AKT into the structures, and phosphorylates them (pAKT). Given the importance of the PI3K-AKT pathway in GF signaling, CDRs are likely involved in cell growth. Interestingly, some cancer cell lines express CDRs. We hypothesized that CDRs contribute to carcinogenesis by modulating the AKT pathway. In the present study, we identified CDR-expressing cancer cell lines and investigated their cellular functions.

**Methods:**

CDR formation was examined in six cancer cell lines in response to epidermal growth factor (EGF) and insulin. The morphology of the CDRs was characterized, and the related signaling molecules were observed using confocal and scanning electron microscopy. The role of CDRs in the AKT pathway was studied using biochemical analysis. The actin inhibitor cytochalasin D (Cyto D) and the PI3K inhibitor TGX221 were used to block CDRs.

**Results:**

GF treatment induced CDRs in the hepatocellular carcinoma (HCC) Hep3B cell line, but not in others, including HCC cell lines HepG2 and Huh7, and the LO2 hepatocyte cell line. Confocal microscopy and western blot analysis showed that the PI3K-PIP3-AKT pathway was activated at the CDRs and that receptor proteins were recruited to the structures. Cyto D and TGX221 completely blocked CDRs and partially attenuated GF-induced pAKT. These results indicate that CDRs regulate the receptor-mediated PI3K-AKT pathway in Hep3B cells and the existence of CDR-independent pAKT mechanisms.

**Conclusions:**

Our results showed that CDRs modulate the AKT pathway in Hep3B cells. Since CDRs were not observed in other HCC and hepatocyte cell lines, we propose that CDRs in Hep3B would determine the carcinoma characteristic of the cell by aberrantly triggering the AKT pathway. Signaling molecules involved in CDR formation are promising therapeutic targets for some types of HCC.

**Video abstract**

**Supplementary Information:**

The online version contains supplementary material available at 10.1186/s12964-022-00911-6.

## Background

Circular dorsal ruffles (CDRs) are large-scale, rounded membrane ruffles mainly induced by stimulation with growth factors (GF), such as platelet-derived growth factor (PDGF), hepatocyte growth factor (HGF), and epidermal growth factor (EGF) [1, 2]. After GF stimulation, the plasma membrane was evoked on the dorsal surface to form round ruffles as CDRs. Similar to other membrane ruffles, cytoskeletal mechanisms and related small GTPases govern CDR formation. Immunofluorescence staining showed that CDRs contain actin and their polymerization proteins, such as cortactin and neuronal Wiskott-Aldrich syndrome protein (N-WASP) [3, 4]. Depletion of these proteins blocks CDR formation [4]. The involvement of other cytoskeletal proteins such as paxillin[5, 6] and vinculin [7] has also been demonstrated. The microtubule polymerization inhibitor nocodazole blocks CDR formation [8]. The small GTPases Rac1, Ras, and Rab5a have been observed at CDRs, and their interactions during formation have been studied [9–12]. Overexpression of the active form of Ras in wild-type Rab5a induced CDRs [9]. Rab5a-induced CDR was blocked by the co-expression of the negative Rac form [10].

Besides cytoskeleton and small GTPases, phosphatidylinositol 3-kinase (PI3K) and the product phosphatidylinositol (3,4,5)-triphosphate (PIP3) are also involved in CDR formation. The PI3K inhibitors wortmannin [13] and LY2940002 [11, 14] inhibit CDR formation. Furthermore, the generation and accumulation of PIP3 inside the CDRs have been observed [8]. SHIP2 is a lipid phosphatase that dephosphorylates PIP3 to phosphatidylinositol (3,4)-bisphosphate (PIP2) [15, 16]. Localization of SHIP2 at CDRs has been observed, and depletion of the protein attenuated CDR formation [14]. Hasegawa et al. identified SH3 and SYLF domain containing 1 (SH3YL1), the protein that binds to PIP3 at CDRs via the SYLF domain [14]. SH3YL1 depletion inhibits PDGF-induced CDRs, and SH3YL1 interacts with SHIP2. Based on these findings, they proposed that the SH3YL1/SHIP2 complex in CDRs regulates the PIP3/PIP2 balance, triggering the Arf small GTPase pathway [2]. Collectively, previous studies have indicated that cytoskeletal mechanisms, small GTPases, and PI signaling pathways coordinate CDR formation.

Several phenomena have been observed as the cellular function of CDRs. In some cases, CDRs gradually shrink towards the center and function as precursors for macropinocytosis, large-scale endocytosis [7, 8]. Further, CDRs internalize EGF receptors [17, 18]. We have been studying the cellular function of PIP3 generated inside CDRs as an upstream signaling molecule of AKT protein kinase [8, 19]. The pleckstrin homology (PH) domain of AKT interacts with PIP3. Inactive AKT is localized in the cytosol. After GF stimulation, activated PI3K generates PIP3 at the plasma membrane, and AKT is recruited to the membrane via the PH-PIP3 interaction. AKT is phosphorylated and activated at the membrane by kinases phosphoinositide-dependent kinase-1 (PDK1) and mTOR complex 2 (mTORC2) [20, 21]. Thus, recruitment of AKT to the plasma membrane is necessary for its phosphorylation (pAKT) and activation. We observed that PIP3 generation in response to EGF stimulation recruited AKT to CDRs and that inhibition of CDR formation inhibited EGF-induced pAKT formation [8]. Based on these findings, we propose that CDRs can be utilized as signaling platforms for the AKT pathway [8, 19]. Given that the PI3K-AKT pathway is a canonical growth factor signaling pathway, CDRs would be involved in the molecular mechanism of cell growth.

Although the physiological relevance of CDRs is unclear, they have been observed in some types of cancer cells. EGF treatment induces CDRs in the human pancreatic cancer PANC1 [17] and the mouse epithelial tumor Mgat5 cell lines [22]. Localization of EGF receptor (EGFR) in CDRs was observed in both cell lines. The human breast cancer SK-BR-3 cell line induces CDRs in response to trastuzumab, a monoclonal antibody that targets human EGFR 2 (HER2) [23]. PDGF and growth arrest-specific 6 (GAS6) proteins induce CDRs in mouse melanoma 2054E [24] and human glioblastoma LN299 cells [7], respectively. CDRs in Mgat5, SK-BR-5, and LN299 cells recruit cortactin [7, 22, 23]. A recent study has shown that CDR-promoted macropinocytosis contributes to focal adhesion turnover in LN299 cells [7].

In this study, we investigated the role of CDRs in cancer cells. Human hepatocellular carcinoma (HCC) ranks fifth in incidence rate, third in mortality, and is one of the most severe complications resulting from chronic liver diseases [25]. More than 10 human HCC cell lines have been established as in vitro models [26]. For instance, HepG2 and Hep3B cells were derived from a 15-year-old Caucasian male and an 8-year-old African American male, respectively [25, 27]. Huh7 cells were derived from a 53-year-old Japanese man. Ever since, the differences in these HCC cell lines have been extensively studied [25, 28–32]. We demonstrate that Hep3B, but not Huh7, HepG2, and the human hepatocyte LO2 cell line, induced CDRs after GF stimulation. Imaging and biochemical analyses showed that both EGF and insulin receptors are located at CDRs to activate the PI3K-PIP3 pathway. Moreover, we observed that pAKT and SH3YL1 were localized to CDRs. We also identified a dominant role for PI3Kβ in CDR formation. These results indicate a functional connection between CDRs and receptor-mediated PI3K signaling pathways in Hep3B cells, and strongly suggest a pathological role for CDRs in HCC.

## Methods

### Reagents, antibodies, and plasmid

Recombinant human EGF and human insulin were purchased from Peprotech (AF-100–15) and AbMole (M9194), respectively. The p110α inhibitor A66 (M1819) and the p110β inhibitor TGX221 (M1795) were obtained from AbMole. Cytochalasin D (abs44058674) was from Absin. The protease inhibitor cocktail (04,693,159,001) was purchased from Roche. Anti-AKT (#9272), anti-pAKT (473) (#4060), anti-ERK1/2 (#4376), and anti-pERK1/2 (#4695) antibodies for western blot analysis were purchased from Cell Signaling Technology. Anti-Rab5A(#2143S), anti-AKT(#2920), and anti-insulin receptor β (#23,413) antibodies used for immunofluorescence staining were obtained from Cell Signaling Technology. Anti-RIN1 (16,388-1-AP) and anti-EGFR (18,986-1-AP) antibodies for immunofluorescence staining were obtained from Proteintech. The anti-cortactin (A9518), anti-N-WASP (A2576), anti-p110α (A0265), and anti-p110β (A0928) antibodies for immunofluorescence staining were obtained from ABclonal. Anti-SH3YL1 antibody (NBP1-84,133) for immunofluorescence staining was obtained from Novus. Rhodamine-phalloidin (RM02835) was obtained from Abclonal. The mounting medium with DAPI (ab104139) was from Abcam. The plasmid PH-Btk-GFP (Addgene, #51,463) was used to express GFP-BtkPH.

### Cell culture, inhibitor treatment, and transfection

Hep3B, HepG2, and BxPC-3 cells were purchased from Tongpai Biotechnology Co., Ltd. (Shanghai, China). Huh7, MCF-7, MDA-MB-231, and LO2 cells were purchased from Hunan Fenghui Biotechnology Co., Ltd (Changsha, Hunan, China). Hep3B, Huh7, HepG2, LO2, and MDA-MB-231 cells were cultured in Dulbecco’s modified Eagle’s medium (DMEM, Gibco, C12430500BT) supplemented with 10% fetal bovine serum (FBS, Gibco, 10,099-141C), penicillin (Shanghai Yuanye Bio-Technology Co., Ltd, B25911), and streptomycin (Sangon Biotech, A610494-0050). MCF-7 and BxPC-3 cells were cultured in RPMI Medium 1640 (Gibco, C11875500BT) with 10% FBS and penicillin/streptomycin. To avoid mycoplasma contamination, cells were treated with prophylactic plasmocin (InvivoGen, ant-mpp), according to the manufacturer’s instructions. For inhibitor treatments, cells were pretreated for 20 min with A66 (6 μM) and 30 min with TGX221 (4 μM) or cytochalasin D (10 μM). Lipofectamine 2000 (11,668,019; Thermo Fisher) was used for transfection according to the manufacturer’s protocol. Plasmid PH-Btk-GFP was purified using the TIANpure Midi Plasmid Kit (TIANGEN #DP107-02).

### Circular dorsal ruffle (CDR) assay

Hep3B cells were cultured overnight on coverslips in low-glucose DMEM (Gibco C11885500BT) without FBS. Cells were stimulated with 160 nM EGF or 100 nM insulin (3 min) and then fixed in fixation buffer A (4% paraformaldehyde in PBS, pH 7.4) at room temperature for 20 min. The fixed cells were washed thrice with TBST (50 mM Tris, 150 mM NaCl, 0.1% Tween 20, pH 7.6) for 10 min at room temperature. The cells were permeabilized in 0.1% Triton X-100 in PBS for 5 min and incubated in blocking buffer (TBST, 1% BSA) for 30 min at room temperature. To identify CDRs, actin was stained with rhodamine-phalloidin. The dye was diluted at 1:100 in the blocking buffer and incubated with the samples for 1 h at room temperature. The samples were washed three times with TBST for 10 min at room temperature and mounted for microscopy. The frequency of cells showing CDRs was determined as previously described [8]. Cells were randomly selected, and the number of cells with CDRs was counted (more than 800 cells per condition, more than three independent experiments). Frequency was calculated as follows: (number of cells with CDR)/(total number of cells observed).

### Immunofluorescence staining and confocal microscopy

Cells were cultured overnight on coverslips in low-glucose DMEM without FBS. After stimulation with EGF or insulin, cells were fixed in fixation buffer A for 20 min at room temperature. For immunofluorescence (IF) staining of AKT and pAKT, the cells were permeabilized in freshly prepared 0.2% saponin in TBST for 15 min at room temperature. For IF staining, cells were permeabilized in 0.1% Triton X-100 in PBS for 5 min and then incubated in blocking buffer for 30 min at room temperature. All antibodies were diluted 1:50 in blocking buffer and incubated with samples overnight at 4 °C for primary antibody treatment. The samples were washed with TBST (three times for 10 min at room temperature). Anti-rabbit IgG Alexa Fluor 488 (Abcam150081) and anti-mouse IgG Alexa Fluor 594 antibodies (Abcam150120) were diluted to 1:500 in the blocking buffer and incubated with the samples for 2 h at room temperature as a secondary antibody treatment. The samples were washed three times with TBST for 10 min at room temperature and then mounted. A Leica TCS SP5 confocal microscope was used at the Core Facility of the College of Life Sciences, Nankai University, China.

### Scanning electron microscopy samples and observation

Hep3B cells were cultured on coverslips with collagen (Type I solution from rat tail, Sigma C3867) and fixed in fixation buffer B (2.5% glutaraldehyde, 0.18 M Na_2_HPO_4_, 0.019 M KH_2_PO_4_, pH 7.2) after stimulation. The samples were submitted to Yimingfuxing Bio (Beijing, China) for embedding, according to standard procedures. A field emission scanning electron microscope (Apreo S LoVac, Thermo Fisher) at the Central Laboratory of Nankai University was used for observations.

### Measurement of CDR area

The area of the CDR was analyzed using ImageJ software. To identify CDRs, a polygon selection tool was used to drag a polygon around the structures. The selected areas were then activated and measured using the Analyze > Set measurement tool. To compare the difference between the areas of CDR-induced EGF and insulin, the resulting values were presented as arbitrary units (AU), wherein the average area of EGF-induced CDRs was 1.0. A two-tailed Student’s t-test was used for statistical analysis.

### Cell lysates and western blotting

Cell lysates were prepared as previously described [8]. Briefly, after the assays, the cells were lysed in cold lysis buffer (40 mM HEPES pH 7.5, 120 mM NaCl, 1 mM EDTA, 10 mM pyrophosphate, 10 mM glycerophosphate, 1.5 mM Na_3_VO_4_, 0.3% CHAPS, and a mixture of protease inhibitors) for 10 min. The lysates were centrifuged at 13,000 g for 15 min at 4 °C. The supernatant was mixed with 5 × sodium dodecyl sulfate–polyacrylamide gel electrophoresis (SDS-PAGE) sample buffer (GenStar, #E153) and boiled for 5 min. The samples were subjected to SDS-PAGE and western blotting with the indicated antibodies.

### Quantification

To quantify the CDRs, the frequency of cells containing CDRs was determined from images of more than 800 cells per condition. The average and standard errors of the frequencies were calculated from at least three independent experiments. One-way ANOVA was used for statistical analysis. The results from at least three independent experiments were used to quantify western blot data. The intensities of pAKT and AKT were measured using ImageJ software to calculate the pAKT/AKT ratio, and the values are presented as arbitrary units (AU). Two-tailed Student’s t-test (Fig. 5 A and D, Additional file 1: Figs. S1 C and S2 C), one-tailed Student’s t-test (for Fig. 5 C and D), or one-way ANOVA (for Fig. 7 B and D, Additional file 1: Fig. S4 B and D) were used for statistical analysis.

## Results

### Growth factor stimulation induces CDRs in human hepatocellular carcinoma (HCC) Hep3B cells

**Fig. 1:**
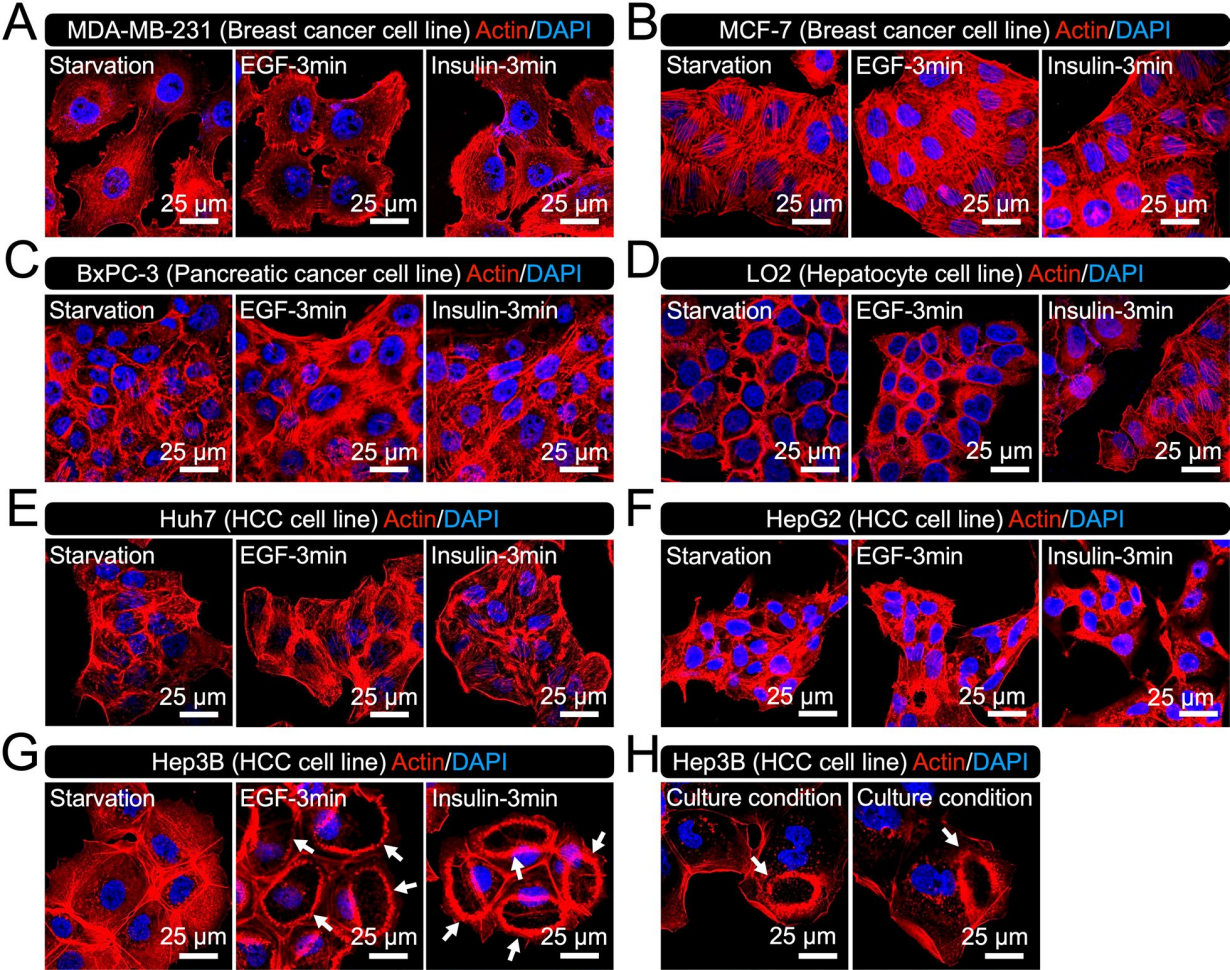
Growth factor stimulation induces CDRs in Hep3B cells. **A**–**G** Representative confocal images of actin in MDA-MB-231 (**A**), MCF-7 (**B**), BxPC-3 (**C**), LO2 (**D**), Huh7 (**E**), HepG2 (**F**), and Hep3B (**G**) cell lines after EGF (160 nM) or insulin (100 nM) treatment (3 min). Cells were starved overnight and stimulated by the ligands. CDRs (arrows) were identified in Hep3B but not in other cell lines. **H** Representative confocal images of actin in Hep3B under culture condition. CDRs (arrows) were occasionally identified. Scale bars: 25 μm

We tested six well-known cancer cell lines for their ability to express CDRs following GF stimulation. Since CDRs were observed in the breast cancer SK-BR-3 cell line and pancreatic cancer PANC1 cell line, two breast cancer cell lines, MDA-MB-231 (Fig. 1A) [33] and MCF-7 (Fig. 1B) [34], and one pancreatic cell line, BxPC-3 (Fig. 1C) [35], were tested. To determine whether CDRs were observed in the HCC cell lines, Huh7 (Fig. 1E) [36], HepG2 (Fig. 1F) [25, 27], and Hep3B (Fig. 1G) [25, 27] were used. The hepatocyte LO2 cell line (Fig. 1D) [37] was also prepared as a control. Among them, we found that only Hep3B cells radically expressed CDRs after EGF treatment (Fig. 1G, EGF, arrows). This was also observed after insulin stimulation (Fig. 1G, Insulin, arrows). Quantitative analysis showed that the insulin-induced CDRs were smaller than those induced by EGF (Additional file 1: Fig. S1). Interestingly, although rare, we observed CDRs in Hep3B cells under culture conditions (Fig. 1H, arrows). These results prompted us to characterize the CDRs in Hep3B cells.

**Fig. 2:**
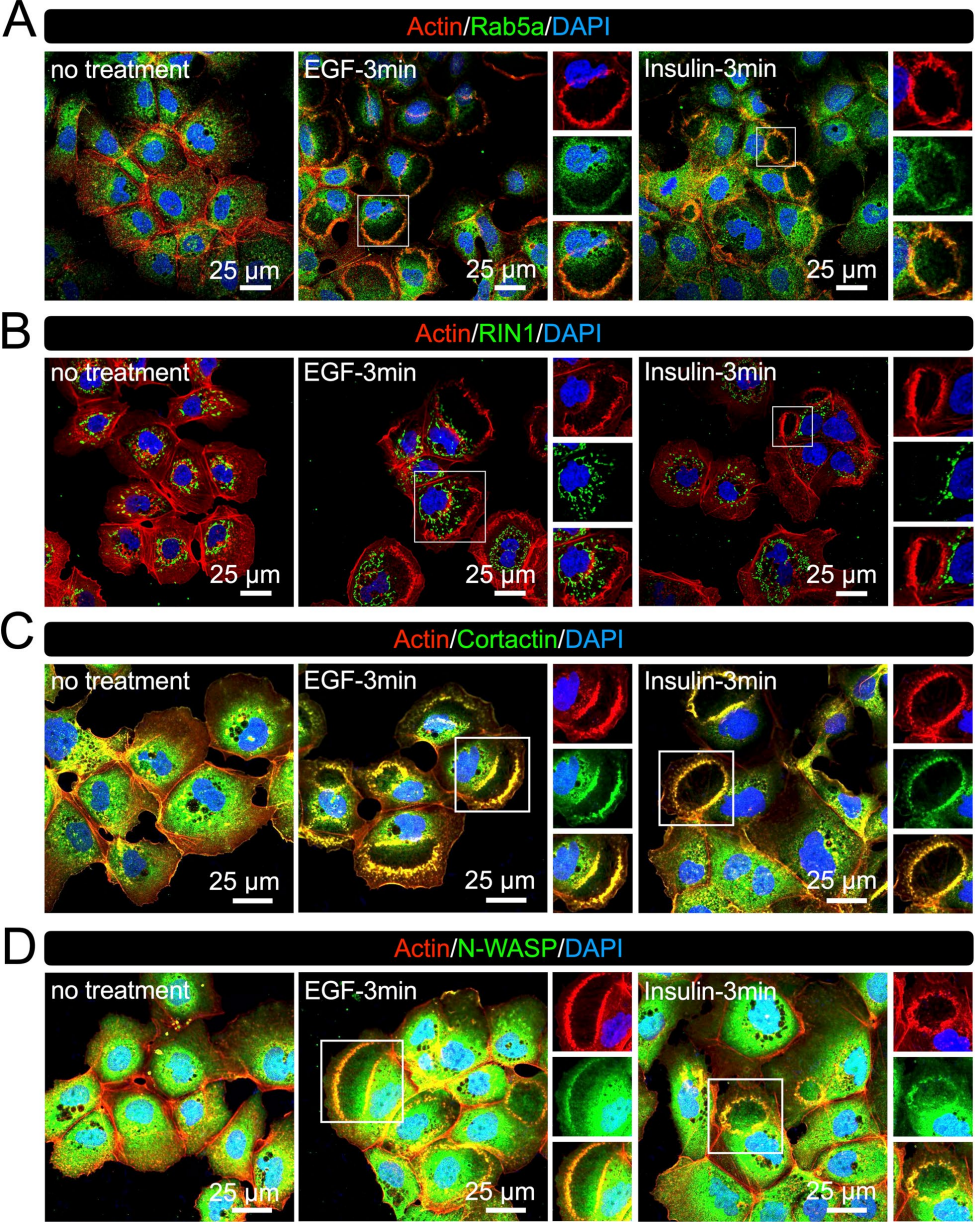
Rab5a, cortactin, and N-WASP are localized at CDRs in Hep3B cells. Representative confocal images of actin-Rab5a (**A**), actin-RIN1 (**B**), actin-cortactin (**C**), and actin-N-WASP (**D**) after EGF or insulin treatment (3 min). Rab5a, cortactin, and N-WASP, but not RIN1, were observed at CDRs (magnified images). Scale bars: 25 μm

The small GTPase Rab5a is recruited to CDRs [9, 10] and can be used as a marker. To confirm that the induced rounded membrane ruffles were CDRs, we localized Rab5a by IF staining. Confocal microscopy clearly showed that Rab5a was located in CDRs induced by EGF and insulin (Fig. 2A). The Ras and Rab interactor 1 (RIN1) protein activates Rab5a as a guanylate exchange factor in GF signaling [38]. However, we did not observe RIN1 at CDRs (Fig. 2B), suggesting that Rab5a is not activated at CDRs or is likely activated by other pathways. The actin polymerization proteins cortactin and N-WASP have been implicated in CDR formation [3, 4], and we observed these proteins at the CDRs in Hep3B cells (Fig. 2C and D).

**Fig. 3:**
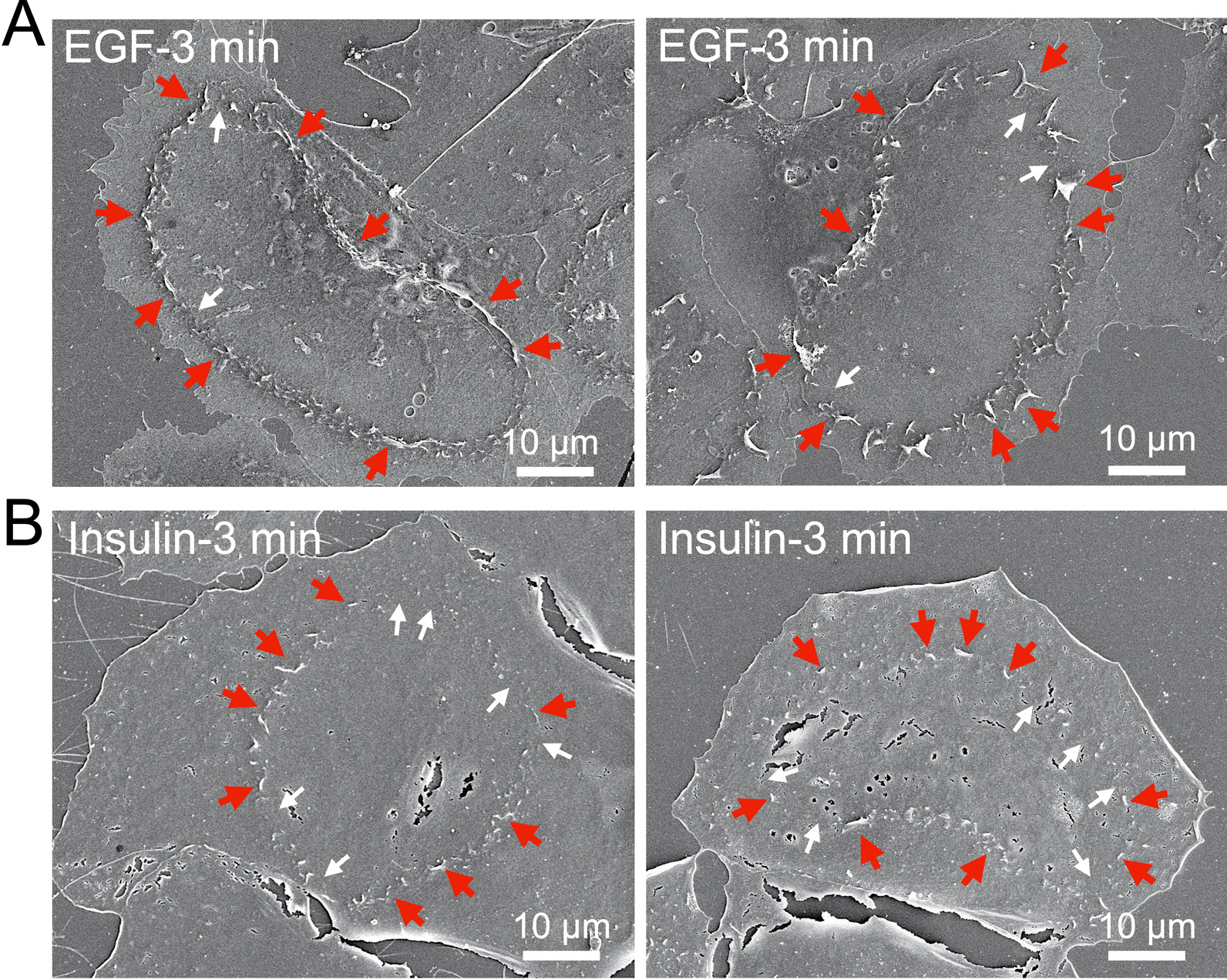
SEM images of growth factor-induced CDRs in Hep3B cells. Representative images of advanced SEM showing CDRs induced in Hep3B cells by EGF (**A**) and insulin (**B**) treatment (3 min). Lamellipodia (red arrows) are vertically evoked from the surface of cells to form CDRs, whereas the structures are disconnected at several locations (white arrows). Scale bars: 10 μm

We further characterized the CDRs in Hep3B cells by SEM analysis (Fig. 3 and Additional file 1: Fig. S2), which revealed that lamellipodia were vertically evoked from the surface of EGF-stimulated cells (Fig. 3A and Additional file 1: Fig. S2A, red arrows). The structure was rounded but disconnected at several locations (Fig. 3A and Additional file 1: Fig. S2A, white arrows). Compared to the EGF-stimulated CDRs, the lamellipodia structures induced by insulin were smaller and thinner (Fig. 3B and Additional file 1: Fig. S2B, red arrows), and the disconnected parts were evident (Fig. 3B and Additional file 1: Fig. S2B, white arrows). Similar to the quantification results obtained using IF staining images (Additional file 1: Fig. S1), we confirmed that the insulin-induced CDRs observed by SEM were smaller than those induced by EGF (Additional file 1: Fig. S2C).

### CDRs are involved in GF-induced AKT phosphorylation (pAKT) in Hep3B

**Fig. 4:**
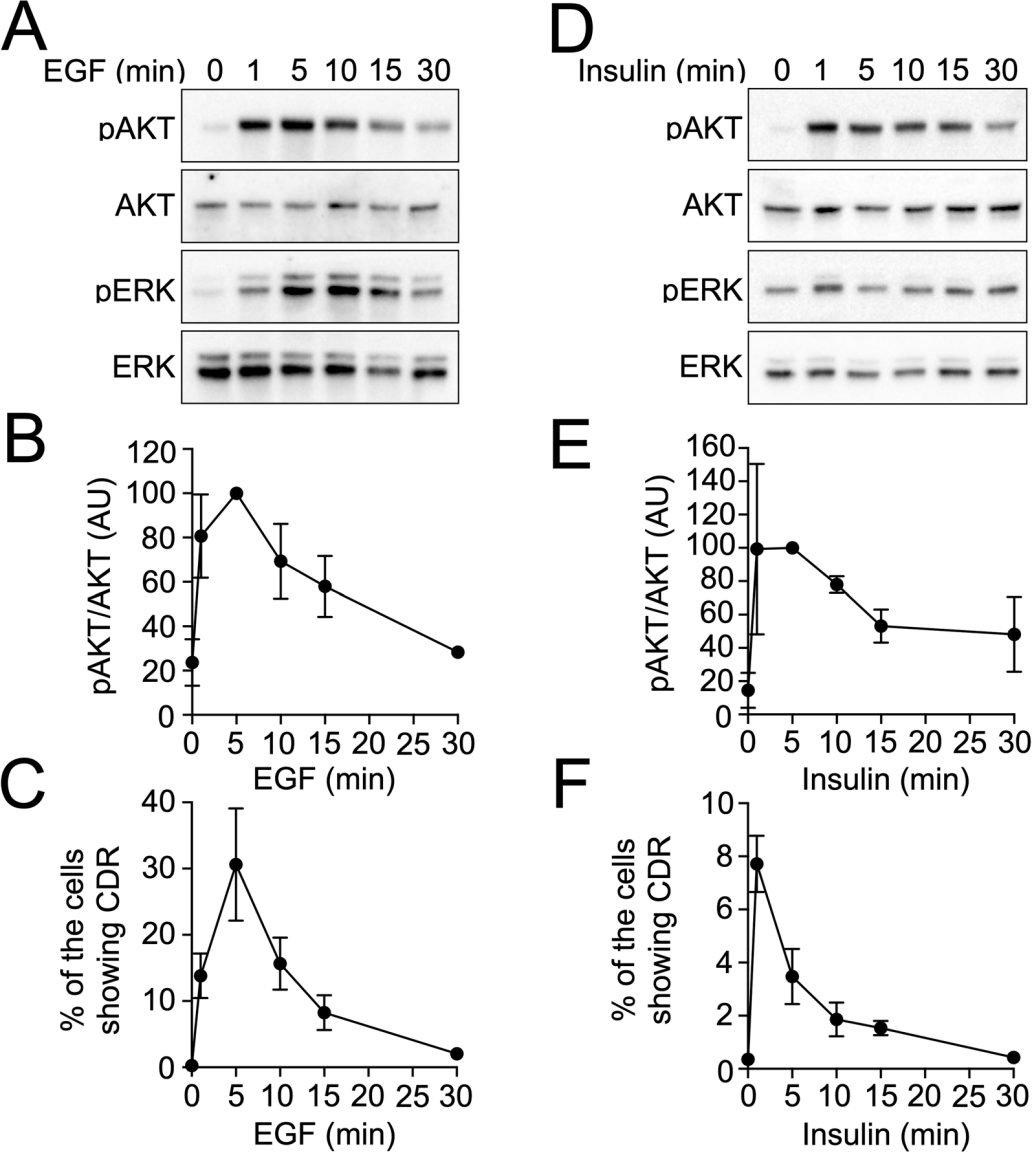
Correlation between AKT phosphorylation and CDR formation in Hep3B cells after growth factor stimulation. **A** and **D** Time course of signaling in response to EGF (160 nM) (**A**) and insulin (100 nM) (**D**). **B** and **E** Quantification of the pAKT/AKT ratio at the indicated times after EGF (**B**) or insulin (**E**) stimulations from three independent experiments. **C** and **F** The frequency of cells showing CDRs at the indicated times after EGF (**C**) or insulin (**F**) stimulations from three independent experiments

Recruitment of AKT to CDRs is critical for pAKT at the plasma membrane of mouse embryonic fibroblasts (MEFs) [8]. Thus, we next investigated the molecular functions of CDRs in Hep3B cells towards pAKT formation. Biochemical analysis showed that EGF stimulation induced pAKT within 1 min; the signal reached its peak at 5 min and then decreased by 30 min (Fig. 4A and B). ERK phosphorylation (pERK) was detected as a control, and the peak was between 5 and 10 min after stimulation. Meanwhile, imaging analysis showed that CDRs were induced within 1 min after stimulation, maximal CDR formation was observed after 5 min, and decreased over the next 25 min (Fig. 4C). Therefore, the time course of pAKT and CDR formation are strongly correlated in EGF-stimulated Hep3B cells. A similar pattern was observed in insulin-stimulated Hep3B cells (Fig. 4D–F). Notably, EGF and insulin induced pAKT formation in Huh7, HepG2, and LO2 cells (Additional file 1:Fig. S3); however, CDRs were not observed in these cells during the time course (not shown), suggesting that the formation of CDR is one of the unique cellular responses of Hep3B cells in the context of the GF signaling.

**Fig. 5:**
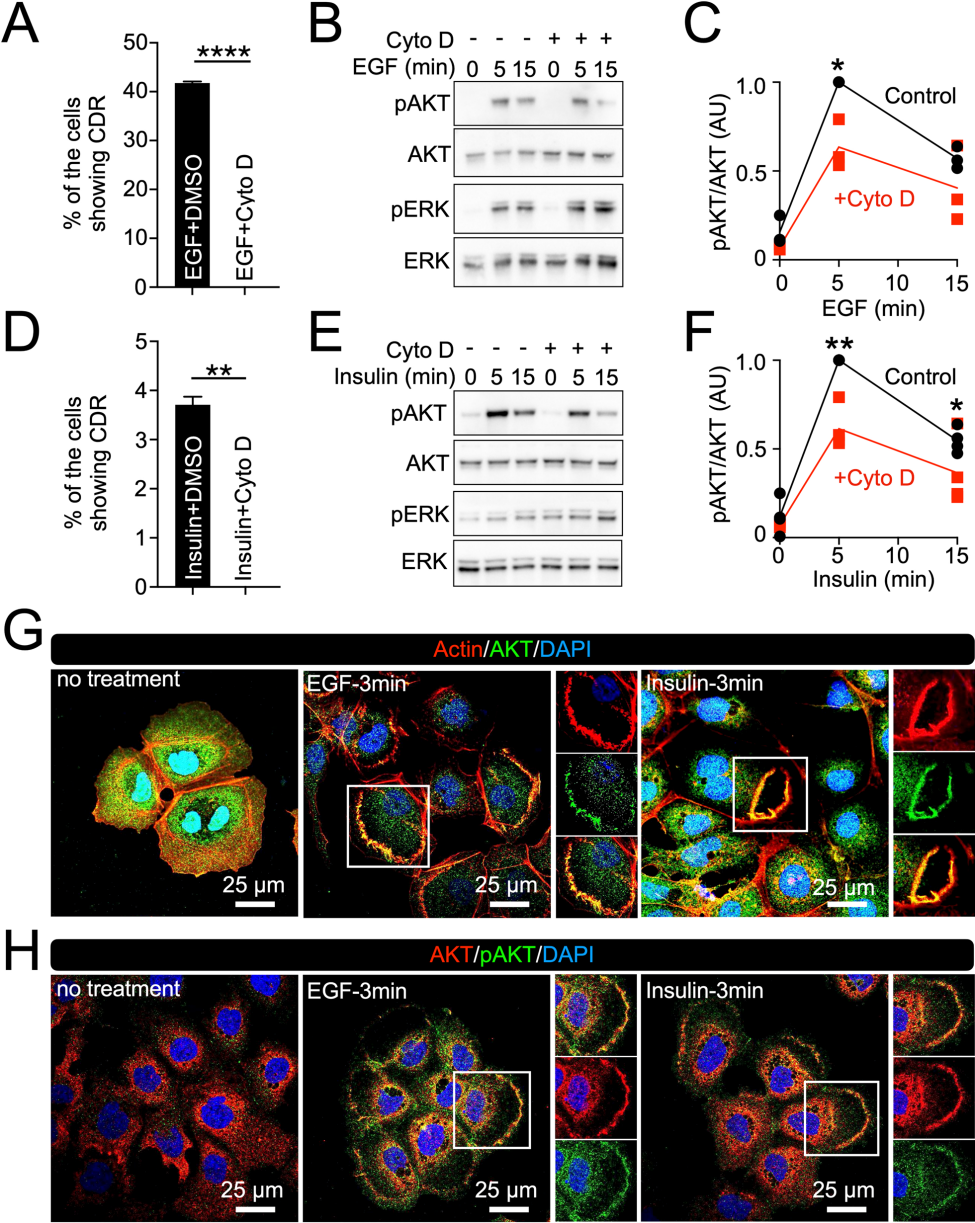
AKT is phosphorylated at CDRs in Hep3B cells. **A** and **D** Cytochalasin D (Cyto D) treatment completely blocked CDR formation in response to EGF (**A**) and insulin (**D**). **P < 0.01, ****P < 0.0001, by two-tailed Student’s t-test. **B** and **E** Cyto D attenuated AKT phosphorylation (pAKT) induced by EGF (**B**) or insulin (**E**). Cyto D did not affect ERK phosphorylation (pERK). **C** and **F** Quantification of pAKT/AKT ratios at the indicated times after EGF (**C**) and insulin (**F**) stimulations without (black) or with (red) Cyto D treatment from three independent experiments. Results are indicated as arbitrary units (AU). *P < 0.05, **P < 0.01, by one-tailed Student’s t-test. **G** and **H** Representative confocal images of actin-AKT (**G**) and AKT-pAKT after EGF or insulin treatments (3 min). A strong AKT signal (green) was observed at CDRs identified by actin staining (red) (**G**, enlarge images). Enlarged images of (**H**) show co-localization of AKT (red) and pAKT (green) at CDRs

CDRs are regulated by actin cytoskeleton dynamics [1, 2]. We utilized the actin polymerization inhibitor cytochalasin D (Cyto D) to determine whether blocking CDR formation affects pAKT formation. Cyto D treatment completely abolished CDR formation in response to EGF and insulin (Fig. 5A and D). Biochemical analysis indicated that pAKT induced by GF treatment was attenuated by Cyto D treatment, whereas pERK was not affected (Fig. 5B, C, E, and F). These results strongly suggested that CDRs are required for GF-induced pAKT in Hep3B cells. The results also suggest that pAKT can be induced independently in CDRs since the inhibitor blocked GF-induced pAKT by approximately 50% (Fig. 5C and F).

We performed confocal microscopy to test whether AKT is localized and phosphorylated at CDRs. As for Rab5a, cortactin, and N-WASP (Fig. 2), we observed strong AKT signals in actin-positive ring-like structures (Fig. 5G), indicating that AKT was located in CDRs. AKT and pAKT double staining showed that AKT was phosphorylated at CDRs (Fig. 5H). Interestingly, both experiments revealed that AKT and pAKT were detected in the cytosol. Thus, these results suggest that CDRs can be used as signal platforms to localize AKT at the plasma membrane for phosphorylation in Hep3B, and there are CDR-independent mechanisms to induce pAKT in the cytosol.

### CDRs are signal platforms for a GF-induced PI3K pathway in Hep3B

**Fig. 6:**
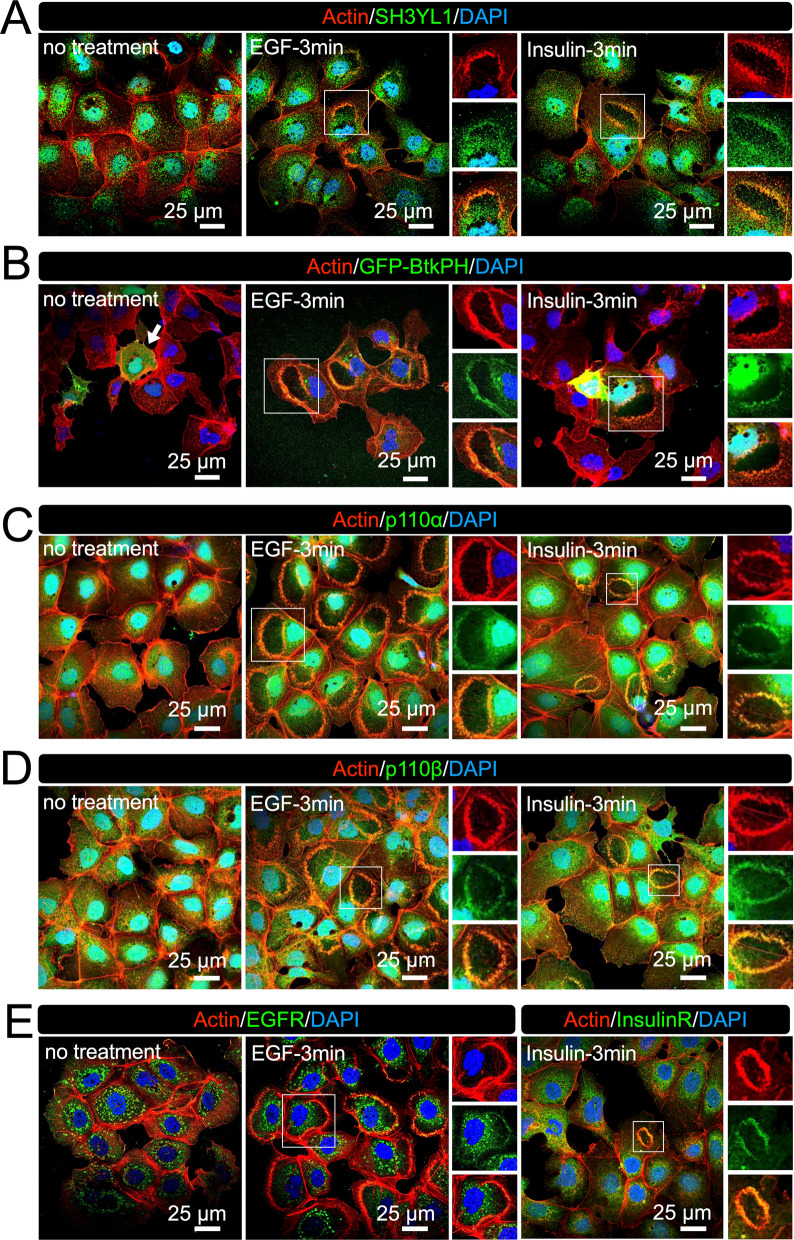
Receptor-mediated PI3K pathway at CDRs in Hep3B cells. **A** Representative confocal images of actin-SH3YL1 showing SH3YL1 is localized at CDRs (enlarge images). (**B** Representative confocal images of actin and GFP-BtkPH. GFP-BtkPH was expressed in Hep3B cells as a probe protein to identify PIP3. Confocal images showed recruitment of GFP-BtkPH to CDRs, indicating that PIP3 was generated at the structures. **C** and **D** Representative confocal images of actin-p110α (**C**) and actin p110β (**D**) showing p110 isoforms are localized at CDRs (enlarge images). **E** Representative confocal images of EGF receptor and insulin receptor. Both receptors are localized at CDRs in Hep3B (enlarge images)

AKT is recruited to the plasma membrane via the interaction of its PH domain with PI3K-generated PIP3 at the membrane [20, 21]. SH3YL1 is a PIP3-binding protein reported to localize to CDRs in mouse embryonic fibroblast NIH3T3 cells [14]. We also observed the recruitment of SH3YL1 to CDRs in Hep3B cells (Fig. 6A). To confirm the generation of PIP3 at the CDRs in Hep3B cells, we used GFP-BtkPH, a well-known probe for PIP3 [8, 39]. After overexpression, the cells were treated with EGF/insulin and fixed for actin staining. Confocal microscopy showed that GFP-BtkPH was mainly localized to the cytosol before GF stimulation (Fig. 6B, arrow) and was recruited to CDRs after stimulation (Fig. 6B, enlarged images). PI3K is a heterodimer consisting of p110 and p85 [40]. Because of the p110 isoforms α and β, there are two PI3K isoforms: PI3Kα and PI3Kβ. Staining for p110α and p110β showed that both were recruited to CDRs (Fig. 6C and D). PI3Ks are recruited to receptor proteins after GF stimulation [40]. We also observed that EGFR and insulin receptor were located at the CDRs after stimulation (Fig. 6E). These results suggest that GF-induced PI3K signal transduction localizes to CDRs in Hep3B cells.

### PI3Kβ regulates CDR-dependent pAKT

**Fig. 7:**
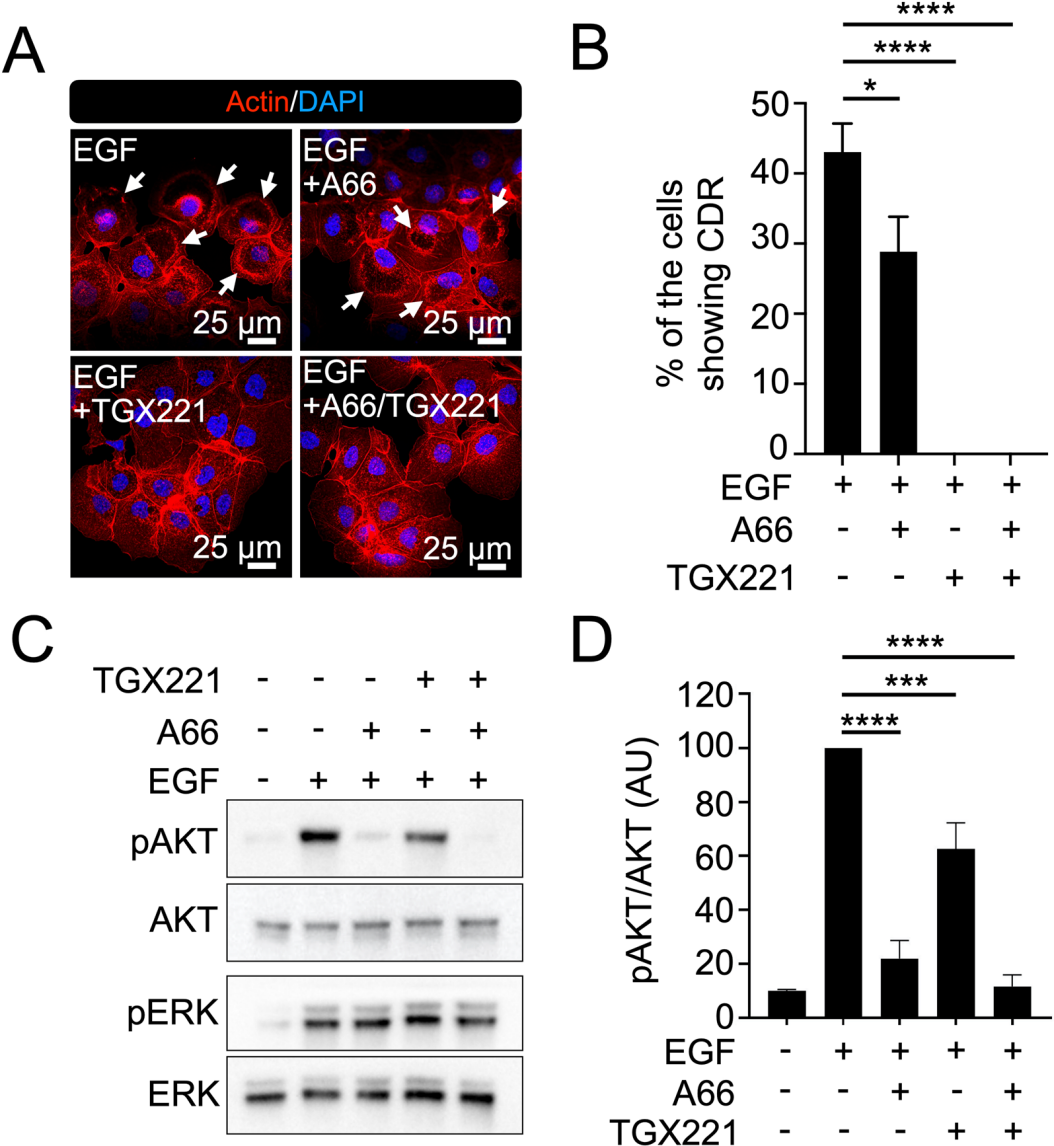
Dominant roles of PI3Kβ in EGF-induced CDR formation. **A** Representative confocal images of actin in Hep3B with or without p110α inhibitor (A66) or/and p110β inhibitor (TGX221) treatment after EGF stimulation. Arrows indicate CDRs. (**B** Quantification of the frequency of CDRs after stimulation by EGF with/without p110 inhibitors from three independent experiments. TGX221 completely blocked CDR. *P < 0.05, ****P < 0.0001, by one-way ANOVA. **C** A66 completely blocked EGF-stimulated (5 min) pAKT. TGX211 attenuated pAKT around 50%. **D** Quantification of pAKT/AKT ratios after EGF stimulation (5 min) with/without p110 inhibitors from 3 independent experiments. Results are indicated as arbitrary units (AU). ***P < 0.001, ****P < 0.0001, by one-way ANOVA

PI3K isoforms have distinct functions in cell growth [40, 41]. To determine the roles of each PI3K in the CDR-dependent PI3K-AKT pathway, we used the p110α-specific inhibitor A66 and p110β-specific inhibitor TGX221. Confocal microscopy revealed that TGX221 completely blocked EGF-induced CDRs, whereas A66 only partially blocked EGF-induced CDRs (Fig. 7A and B). Interestingly, the biochemical analysis showed that A66 almost completely blocked EGF-induced pAKT, whereas TGX221 mildly but significantly attenuated the signal (Fig. 7C and D). Similar results were obtained with insulin (Additional file 1: Fig. S4). These results suggest that PI3Kβ plays a critical role in CDR formation and inhibition of PI3Kβ blocked CDR-dependent, but not CDR-independent, pAKT formation. These results also suggest that PI3Kα plays a significant role in generating pAKT.

## Discussion

In the current study, we screened six cancer cell lines and found that human HCC Hep3B cells exhibited CDRs (Fig. 1). EGF treatment stimulated CDR formation in Hep3B cells (Fig. 1G), as demonstrated in other cell lines, such as PANC1 [17], murine mammary epithelial cancer cells [22], and MEFs [8]. Additionally, we report for the first time that CDRs were also induced in response to insulin treatment (Fig. 1G). CDRs were occasionally observed in Hep3B cells under culture conditions (Fig. 1H). Confocal microscopy showed that the CDR markers, Rab5a [9, 10], cortactin [7, 22, 23], and N-WASP [3], were recruited to the CDRs (Fig. 2). Further, high-resolution SEM revealed that CDRs in Hep3B cells were rounded but disconnected large-scale membrane ruffles (Fig. 3 and Additional file 1: Fig. S2). Notably, the combination of imaging techniques and biochemical analyses revealed that CDRs could be used as signal platforms for the GF receptor-related PI3K-AKT pathway (Figs. 4, 5, 6, 7). There was a strong correlation between pAKT and CDR formation after GF stimulations (Fig. 4). Indeed, the actin polymerization inhibitor cytochalasin D (Fig. 5A–F) and the PI3Kβ inhibitor TGX221 (Fig. 7 and Additional file 1: Fig. S4) completely blocked CDR formation and significantly attenuated GF-induced pAKT in Hep3B cells. Further imaging analysis showed that the GF receptors (Fig. 6E), p110α (Fig. 6C), p110β (Fig. 6D), and AKT (Fig. 5G) were located at the CDRs. Indeed, AKT and pAKT double staining confirmed the colocalization of both signals within CDRs (Fig. 5H). Moreover, recruitments of SH3YL1 and GFP-BtkPH were observed (Fig. 6A and B), suggesting that PIP3 was generated at CDRs. Other HCC cell lines such as Huh7 cells (Fig. 1E) and HepG2 cells (Fig. 1F), together with the hepatocyte LO2 cell line (Fig. 1D), did not form CDRs, rendering CDR as a unique phenotypic feature of Hep3B cells. Altogether, these data suggest that, in contrast with normal hepatocytes, a CDR-dependent AKT phosphorylation may aberrantly occur in Hep3B cells. Although AKT is a primary signaling molecule that regulates multiple cellular functions, this CDR-mediated aberrant activation may disturb normal cell growth and contribute to carcinogenesis.

The cellular functions of CDRs and their physiological relevance have been studied [1, 2]. We and others have shown that CDRs function in macropinocytosis [3, 7, 8, 14, 42, 43]. Recent studies have shown that macropinocytosis is involved in mTORC1 activation [44], and it has been hypothesized that CDRs are also related to growth factor signaling [45]. Notably, we have previously demonstrated that CDRs serve as macropinocytic cups to regulate the AKT-mTORC1 pathway in MEF [8]. Moreover, it was shown that CDRs changed to macropinocytosis and recycle integrin from the cell surface to new focal adhesions [14]. Meanwhile, it has been shown that EGFR is internalized via the transformation process from CDR to macropinocytosis [46], although the role of macropinocytosis is controversial [17]. CDRs have been observed in several types of cancer cells, such as human glioblastoma LN229 [7], mouse melanoma [24], human breast cancer cell line SKBR3 [23], mouse mammary epithelial cancer cells [22], and human pancreatic tumor cells PANC1 [17]. It has been shown that the oncosuppressor protein p53 suppresses PDGF-induced CDRs [47, 48] and that the depletion of the oncoprotein c-Abl inhibits PDGF-induced CDRs [49]. Additionally, a recent study revealed the role of CDRs in cancer cell invasion [7] by their induction in LN229 cells and disassembly of focal adhesions after receptor tyrosine kinase AXL stimulation by GAS6 ligand. Based on these findings, the authors proposed that GAS6-induced CDRs would trigger mesenchymal cell migration by inducing the turnover of focal adhesions. Besides fibroblasts, epithelial cells, and cancer cells, studies also suggest that specific primary cells, such as rat aortic smooth muscle (RASM) cells [47], rat vascular smooth muscle cells [48], and mouse kidney epithelial cells [50, 51], also can form CDRs after GF stimulation. However, CDRs have not yet been observed in vivo.

**Fig. 8:**
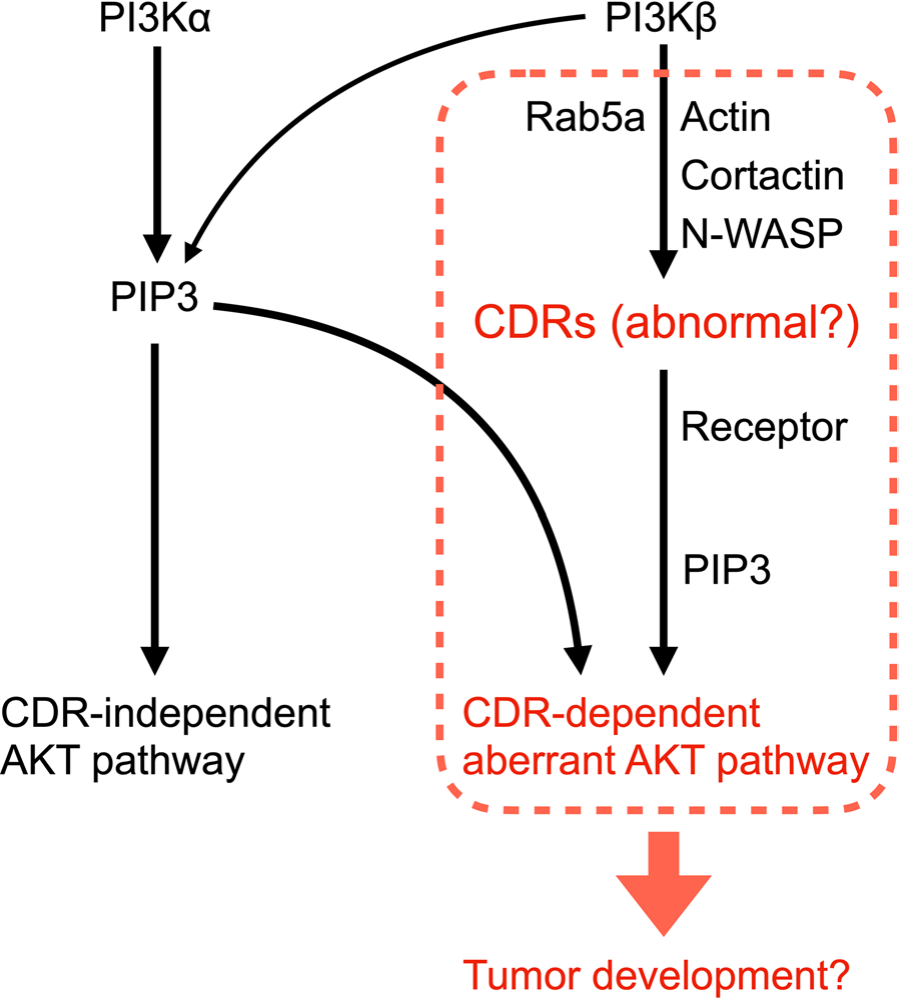
Proposed model of CDR-dependent aberrant AKT pathway in Hep3B cells. Growth factor stimulations abnormally induce CDRs via PI3Kβ in Hep3B. During the process, actin and polymerization proteins such as cortactin and N-WASP are involved. Rab5a is also activated as the downstream signaling molecule of PI3Kβ, although the function is unknown. The abnormal CDRs recruit receptor proteins, which trigger the PI3K pathway. PIP3 is generated at the structures and induces aberrant pAKT formation, leading to tumor development. Meanwhile, PIP3 can be generated outside CDRs mainly by PI3Kα as the normal pathway

Based on our new findings and previous research, we hypothesize that CDRs determine the characteristics of Hep3B cells as carcinoma and proposed a model for the molecular function of CDRs in tumor development (Fig. 8). We propose that Hep3B cells abnormally express CDRs, which trigger an aberrant AKT pathway upon GF stimulation. After stimulation, Hep3B evokes “abnormal” CDRs, wherein the receptor proteins are forced to be located. These receptor proteins recruit PI3Ks to generate PIP3 in the area, prompting downstream signaling pathways, such as pAKT and SH3YL1 recruitment. Rab5a and the actin polymerization proteins cortactin and N-WASP are involved during this process. CDR-dependent pathways would disturb cell metabolism and result in tumor development. Meanwhile, CDR-independent pAKT mechanisms in the cytosol would regulate “normal” GF-signaling pathways.

One interesting question raised by the current research is “why does Hep3B, but not other HCC cell lines or hepatocyte cell lines, induce CDRs?” In other words, which molecular mechanism allows Hep3B cells to express CDRs? Previous reports have shown that the co-expression of active Ras (RasV12) and Rab5a induces CDRs in MEFs [9]. Other studies have shown the recruitment of Rab5a to CDRs in MEFs [9] and HeLa cells [10]. In this study, we observed the recruitment of Rab5a to CDRs in Hep3B cells (Fig. 2A). Ras was overexpressed in Hep3B cells compared to other HCC cell lines [52]. Thus, excessive expression of Ras in Hep3B cells would activate molecular mechanisms involving Rab5a to form CDR. As a critical molecule that interacts with Ras and Rab5a, we predicted that RIN1 would be a suitable candidate since this protein is a Ras effector and works as a Rab5a GEF in the EGF pathway [38]. However, confocal microscopy showed that RIN1 did not localize to the CDRs in Hep3B cells (Fig. 2B), suggesting that the protein has minimal involvement in CDR formation. Ras also activates Rac1 via the Rac-specific GEF Tiam1 [53]. Overexpression of Rab5a induces CDRs in HeLa cells, which are blocked by the co-expression of the dominant negative form of Rac [10]. Further tests are required to ascertain whether the Ras-Tiam1-Rac pathway and Rab5a orchestrate CDR formation independently or cooperatively. We hypothesized that disturbances in cellular functions, such as hyperactivation of growth factor signaling, dysregulated actin polymerization, and mutation of small GTPases, would trigger “abnormal” CDRs formation in Hep3B cells as well as other cancer cells such as LN229, SKBR3, and PANC1.

The mechanism of actin polymerization underlying CDR formation has been extensively studied. As previously reported for N-WASP [3, 50] and cortactin [7, 22, 23], we observed that N-WASP and cortactin were recruited to CDRs in Hep3B cells (Fig. 2C and D). Consequently, the N-WASP inhibitor, wistostatin, blocks GF-induced CDRs [3, 50]. Knockdown of WIP, which interacts with N-WASP, also attenuates GF-induced CDRs [54], similarly to cortactin knockdown [4]. In addition, the well-known actin polymerization proteins WAVE1 and WAVE2 are also recruited to CDRs [13, 55, 56]. The actin cytoskeleton regulator Abi1 protein, a critical component of the WAVE2 complex [57], is also involved in CDR formation, as shown in Abi1-KO MEF cells [55]. Further knock-out experiments of the WAVE1 and WAVE2 proteins in RASM cells showed that only WAVE2 played a role in CDRs formation [47]. The involvement of other cytoskeletal proteins such as paxillin [5–7], vinculin [7], and talin [7] has also been reported. Based on these findings, we presumed that the mechanism involving the cytoskeletal actin indirectly regulates cell growth and cancer development via CDR formation.

A dominant role of PI3Kβ in CDR formation has been observed in NIH3T3 cells [58]. We also observed that the inhibition of PI3Kβ, but not PI3Kα, completely blocked CDRs in Hep3B cells (Fig. 7A–B and Additional file 1: Fig. S4A–B). Rac functions as an upstream signaling molecule of p110β, but not p110α [40, 41]. Therefore, it would be proposed that at least two key pathways modulate CDRs in Hep3B cells: 1) excessive expression of Ras continuously activates Rac via Tiam1, and 2) the Rac-PI3Kβ pathway is accidentally over-activated. Interestingly, an interaction between PI3Kβ and Rab5a has been demonstrated [41, 59–61]. Rab5a-induced CDRs were blocked using the PI3K inhibitor wortmannin [10]. These results indicate complicated interactions between small GTPases and PI signals and suggest that Ras and PI3Kβ play central roles in the network. Focusing on these molecules would be an excellent strategy to identify the molecular mechanism of CDR in Hep3B cells.

In summary, we revealed that the human HCC cell line Hep3B abnormally expresses CDRs. We propose a new concept called “abnormal CDRs,” which trigger tumor development by disturbing the AKT pathway. To date, CDRs have only been observed in vitro using cell lines or primary cells. Owing to the lack of information about both the mechanisms and functions, we concede that the definition of CDRs has not yet been established. Thus, the functional difference between normal and abnormal CDRs is ambiguous. However, it could be presumed that there are different types of CDRs in terms of cellular functions, although morphological behaviors can be observed in the same way. Comparing CDRs in various cell types, especially cancer cell lines and primary cells, could identify the unique characteristics. If CDRs have critical roles in cell growth and differentiation, they can be observed in vivo. Discovering CDRs in tissues is a direct way to establish the definition of normal CDRs.

## Conclusions

Although CDRs have been observed in cancer cells, their molecular mechanisms and functions remain unknown. In the current study, we revealed that CDRs are induced in Hep3B cells as a process to arrange the GF-receptor-mediated PI3K-AKT pathway. Based on these findings, we propose that CDRs in cancer cells are abnormal and trigger aberrant growth factor signaling. Although further studies are required to test this hypothesis, we hypothesize that abnormal CDRs in Hep3B cells contribute to carcinogenesis. In this case, the signaling molecules involved in the molecular mechanism of CDRs would be promising therapeutic targets for some types of HCC.

## Supplementary Information

**Additional file 1**. Figs. S1–S4.

## Data Availability

The datasets presented in this study and the original data are available from the corresponding author upon reasonable request.

## Abbreviations

CDRs: Circular dorsal ruffles
GF: Growth factor
HCC: Hepatocellular carcinoma
EGF: Epidermal growth factor
PDGF: Platelet-derived growth factor
HGF: Hepatocyte growth factor
PI3K: Phosphatidylinositol 3-kinase
PIP2: Phosphatidylinositol (3,4)-bisphosphate
PIP3: Phosphatidylinositol (3,4,5)-triphosphate
Cyto D: Cytochalasin D
pAKT: AKT phosphorylation
pERK: ERK phosphorylation
EGFR: EGF receptor
HER2: Human EGFR 2
GAS6: Growth arrest-specific 6
SEM: Scanning electron microscopy
DMEM: Dulbecco’s modified Eagle’s medium
FBS: Fetal bovine serum
RASM: Rat aortic smooth muscle
