# Supplementary material for: Circular dorsal ruffles disturb the growth factor-induced PI3K-AKT pathway in hepatocellular carcinoma Hep3B cells
Source: Cell Commun Signal. 2022 Jul 7;20:102. doi: 10.1186/s12964-022-00911-6 (PMC9264614; doi:10.1186/s12964-022-00911-6)
Supplement: Supplementary file 2 — Additional file 1. Figs. S1–S4. [file 12964_2022_911_MOESM2_ESM.pdf]

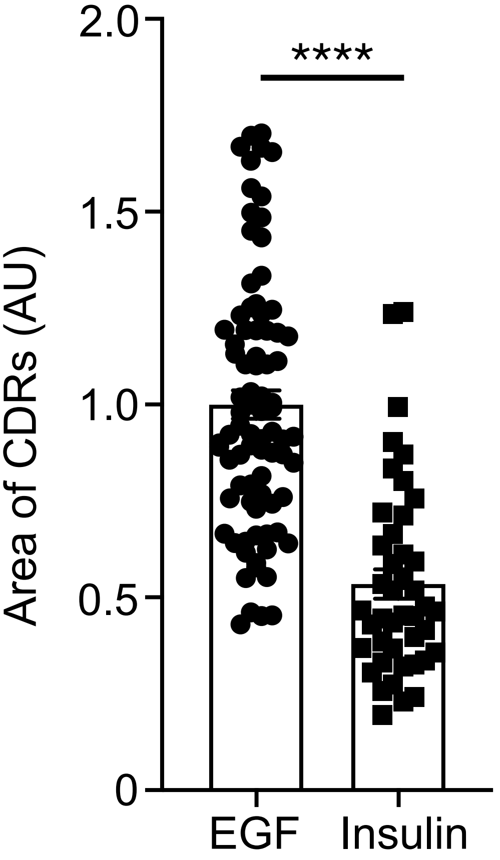

Figure. S1 Quantification of the size of CDRs induced by EGF (n=82) and insulin (n=43) taken with confocal microscopy. The size of CDRs induced by insulin is less than that of EGF. **** $P<0.0001$, by two-tailed Student's t-test.

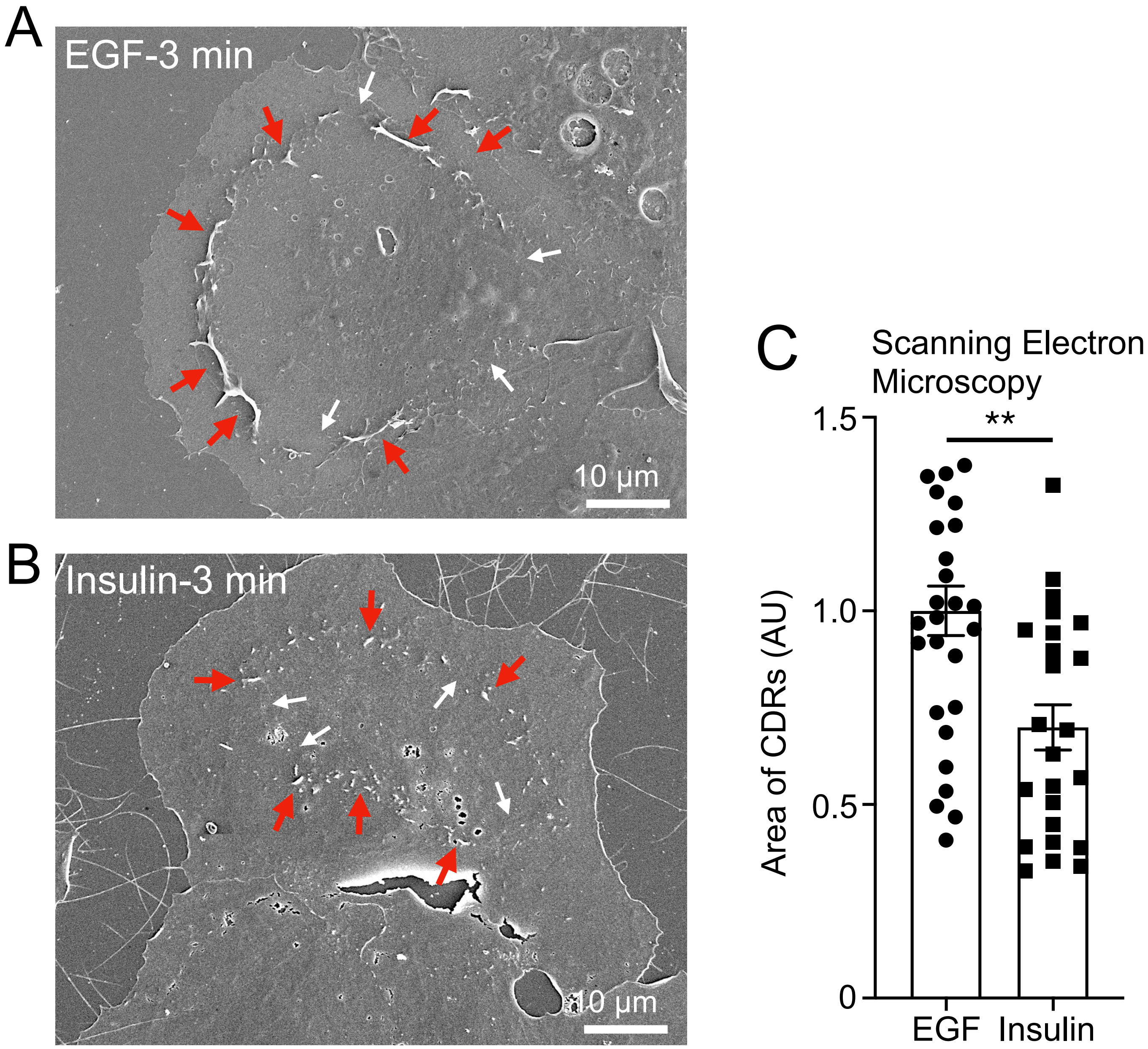

Figure. S2 SEM images of CDRs in Hep3B cells. (A and B) Representative image of advanced SEM image showing EGF (A), and insulin (B) induced CDR formation in Hep3B cells. **(C)** Quantification of the CDR size induced by EGF (n=28) and insulin (n=24) captured by SEM analysis. The size of CDRs induced by insulin is less than that of EGF. **P<0.01, by two-tailed Student's t-test.

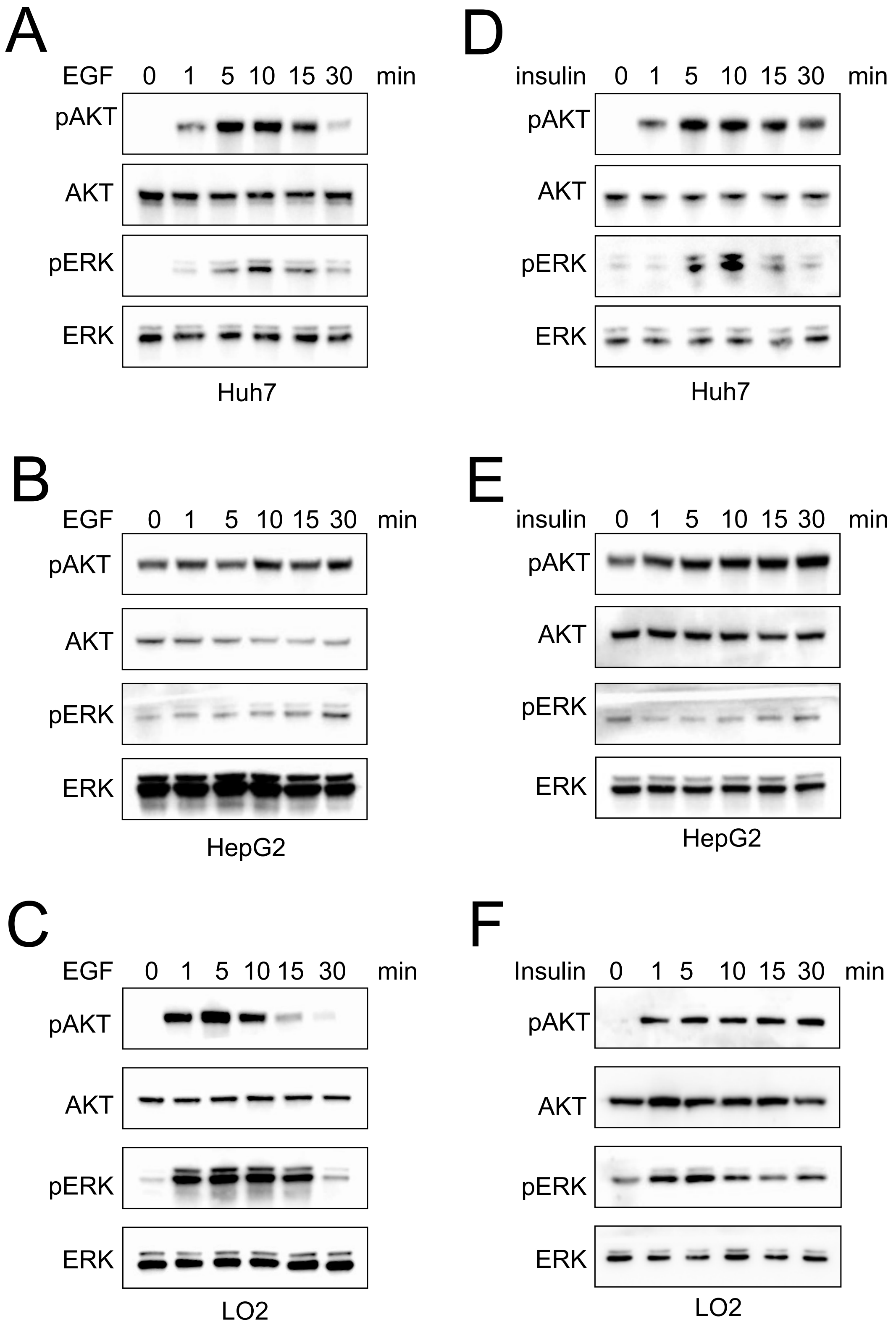

Figure. S3 Time course of signaling in response to EGF (160 nM) (A–C) and insulin (100 nM) (D–F), in Huh7 (A and D), HepG2 (B and E), and LO2 (C and F) cells.

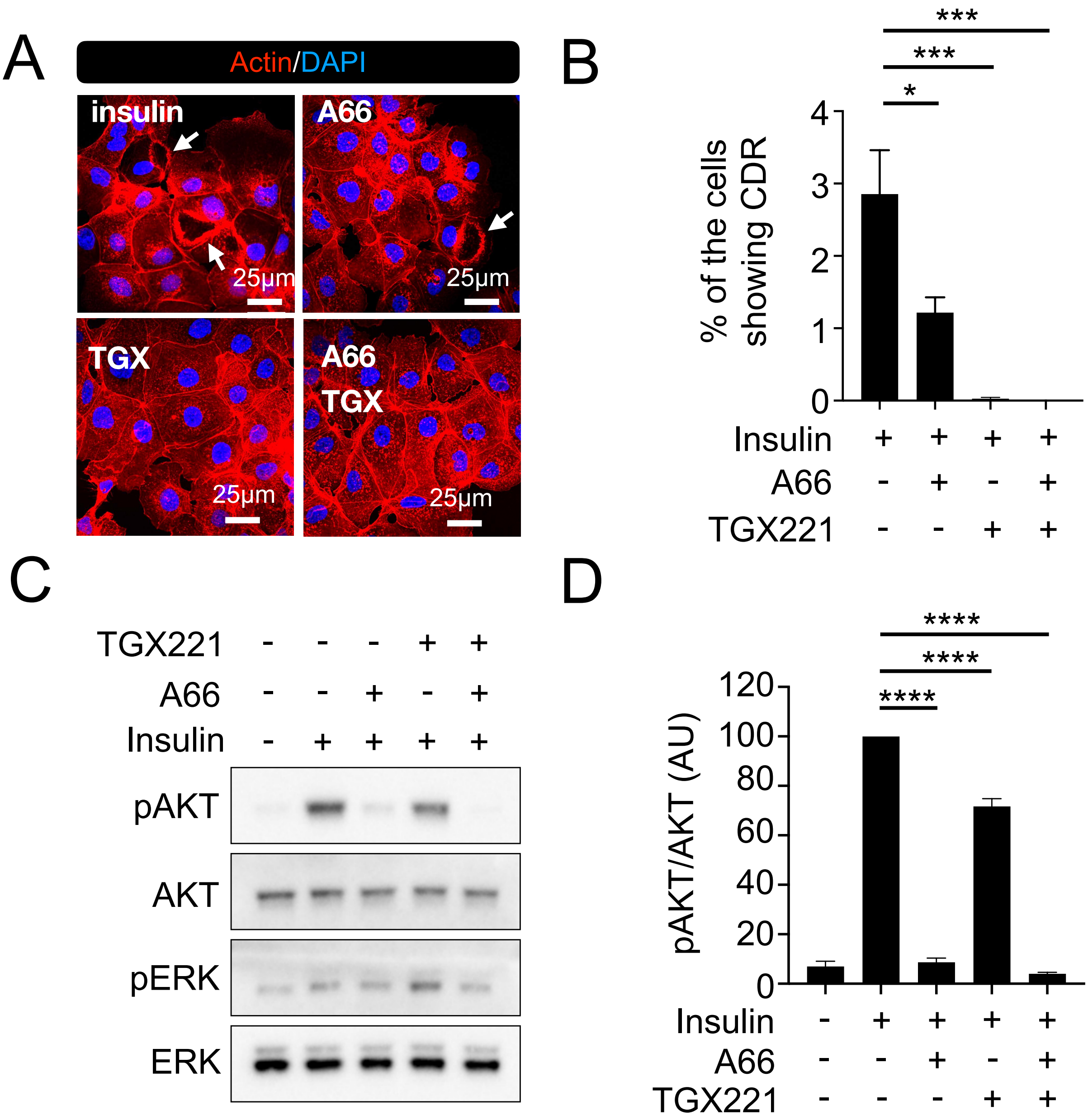

Figure. S4 Dominant role of PI3Kβ in insulin-induced CDR formation. (A) Representative confocal images of actin in Hep3B cells with or without p110α inhibitor (A66) or/and p110β inhibitor (TGX221) treatment after insulin stimulation. Arrows indicate CDRs. **(B)** Quantification of the frequency of CDRs after stimulation by insulin with/without p110 inhibitors from three independent experiments. TGX221 completely blocked CDR. *$P<0.05$, ***$P<0.001$, by one-way ANOVA. **(C)** A66 completely blocked insulin-stimulated (3 min) pAKT, whereas TGX211 attenuated pAKT formation to approximately 50%. **(D)** Quantification of pAKT/AKT ratios after insulin stimulation (3 min) with/without p110 inhibitors from three independent experiments. Results are indicated as arbitrary units (AU). ****$P<0.0001$ by one-way ANOVA.
